# DNA methylation and temperature stress in an Antarctic polychaete, *Spiophanes tcherniai*

**DOI:** 10.3389/fphys.2014.00173

**Published:** 2014-05-05

**Authors:** Adam G. Marsh, Annamarie A. Pasqualone

**Affiliations:** ^1^Center for Bioinformatics and Computational Biology, School of Marine Science and Policy, University of DelawareLewes, DE, USA; ^2^Marine Bioscience, School of Marine Science and Policy, University of DelawareLewes, DE, USA

**Keywords:** epigenetics, DNA methylation, cold acclimation, polychaete, energy metabolism, Antarctica

## Abstract

Epigenetic modifications of DNA and histones are a primary mechanism by which gene expression activities may be modified in response to environmental stimuli. Here we characterize patterns of methyl-cytosine composition in the marine polychaete *Spiophanes tcherniai* from McMurdo Sound, Antarctica. We cultured adult worms at two temperatures, −1.5°C (ambient control) and +4°C (warm treatment), for 4 weeks. We observed a rapid capacity for *S. tcherniai* organismal respiration rates and underlying catalytic rates of citrate synthase at +4°C to return to control levels in less than 4 weeks. We profiled changes in the methylation states of CpG sites in these treatments using an NGS strategy to computationally reconstruct and quantify methylation status across the genome. In our analysis we recovered 120,000 CpG sites in assembled contigs from both treatments. Of those, we were able to align 28,000 CpG sites in common between the two sample groups. In comparing these aligned sites between treatments, only 3000 (11%) evidenced a change in methylation state, but over 85% of changes involved a gain of a 5-methyl group on a CpG site (net increase in methyation). The ability to score CpG sites as partially methylated among gDNA copies in a sample opens up a new avenue for assessing DNA methylation responses to changing environments. By quantitatively distinguishing a “mixed” population of copies of one CpG site, we can begin to identify dynamic, non-binary, continuous-response reactions in DNA methylation intensity or density that previously may have been overlooked as noise.

## 1. Introduction

The emergent field of environmental epigenetics broadly encompasses all heritable changes in gene function and expression that are not associated with a change in underlying genomic DNA sequences (Ho and Burggren, 2010). The epigenome itself represents a dynamic system of chemical controls that impact gene expression at a molecular level (Jaenisch and Bird, 2003). DNA methylation is a primary component of the epigenome and involves the covalent bonding of a methyl group (CH_3_) to the carbon 5 position of a cytosine ring, which forms 5-methyl-cytosine (Feil, 2006).

Unlike the underlying genome which remains largely static across cell types and throughout the course of an individual's lifespan, the methylome is a dynamic system that is influenced by both intrinsic and external environmental signals (Wolffe and Matzke, 1999). Epigenetic modifications of DNA represent an important mechanism through which organisms are able to quickly adjust gene expression in response to changes in environmental conditions including thermal stress. Due to the innate plasticity of DNA methylation of cytosine bases, environmental cues can induce epigenetic shifts in the timing and intensity of gene expression that may contribute to a physiological acclimation response.

Differential methylation patterns at homologous loci may result in functional epialleles that allow organisms to respond to environmental stimuli by expressing alternative phenotypes, which can include behavioral, developmental, morphological, and physiological modifications (Angers et al., 2010). These modified epigenetic profiles enable organisms to maintain performance in the context of an environmental change and facilitate short-term acclimation. A number of studies focusing on both plant and animal models have demonstrated the role of DNA methylation in coordinating the regulation of physiological processes during thermal stress through the epigenetic control of gene expression (Boyko and Kovalchuk, 2008; Pecinka et al., 2010; Correia et al., 2013). Direct correlations between environmental temperature and genome methylation have been described in fish (polar to tropical; Varriale and Bernardi, 2006a) and in temperate reptiles (Varriale and Bernardi, 2006b). Work with invertebrates has revealed the large importance that DNA methylation plays in genome evolution and environmentally driven gene expression shifts (Elango et al., 2009; Gavery and Roberts, 2010; Okamura et al., 2011; Falckenhayn et al., 2013; Glastad et al., 2013; Wang et al., 2013).

The annelid family Spionidae consists of tubicolous polychaetes that inhabit coastal marine sediments (Grube, 1860). Organisms within this genus utilize characteristic prehensile palps originating dorsolaterally from the peristomal segment for construction of individual tubes (Blake and Arnofsky, 1999). These tubes, which can reach lengths of 85 mm and have a hollow 2 mm diameter, are constructed of sediment that has been cemented by mucous. An ecosystem engineer, *S. tcherniai* populations in McMurdo Sound, Antarctica, can form dense tube mats containing over 3000 individuals per square meter (Conlan et al., 2010; Kim et al., 2010). *Spiophanes tcherniai* is characterized primarily as a selective deposit feeder with the capacity for secondary suspension feeding (Oliver and Slattery, 1985; Kim et al., 2010). An analysis of this species' gut constituents indicates a diet composed primarily of amorphous organic material and diatoms (Oliver and Slattery, 1985).

In coastal Antarctic waters, *S. tcherniai* inhabits soft sediments and has been observed from intertidal regions to depths reaching 851 m (Blake, 1983). Among the first species to colonize disturbed environments, this opportunistic species has been observed naturally in environments with varying degrees of physical disturbance, organic enrichment, and chemical pollution (Lenihan and Oliver, 1995; Conlan et al., 2004). The broad distribution and high local abundances of this species in a variety of benthic environments in the Southern Ocean suggest that *S. tcherniai* has a high capacity for acclimating to diverse polar habitats.

Here we identify shifts in methyl-cytosine composition within the genome of the Antarctic marine polychaete *S. tcherniai* in response to an increase in seawater temperature. The high latitude of McMurdo Sound, Antarctica, (77° 51.063′S), makes this polar sea stenothermal at −1.86°C, the freezing point of seawater at 32 psu. A few days each year seawater temperatures may rise above −1.0°C, with −0.5°C being the seasonal high water temperature of recent record (Hunt et al., 2003). As global sea surface temperatures rise, polar regions are projected to evidence some of the largest changes in ecosystem structure because of their long stenothermal history (Moline et al., 2008; Schofield et al., 2010; Doney et al., 2012; Richard et al., 2012). Understanding mechanisms and capacities for surviving increasing temperatures is important in a global context of climate change in polar seas.

## 2. Materials and methods

### 2.1. Polychaete culture and treatments

Live individuals were obtained from sediment collected by SCUBA at the Intake Jetty off of McMurdo Station (77° 51.063′S, 166° 39.867′E). Dense *S. tcherniai* tube mats cover the soft sediment around the jetty, and it is easy for divers to scoop these mats into buckets to be lifted back to the surface. The buckets were immediately transported to the McMurdo Station aquarium facility were the adult tubes could be quickly sieved from the bottom sediments under ambient (−1.5°C) running seawater. The tubes were visually sorted and those containing adult worms were placed into 8 subcultures containing at least 100 individuals each. These adults were maintained at −1.5°C and 32 ppt salinity in the running seawater facility at McMurdo Station. They were fed ground Tetramin® Veggie Fish Flakes (nutritional data available in Marsh et al., 1989) once a week while held in the seawater tables for 2 weeks.

After this initial acclimation period, adult worms were transferred to 11.4 L plastic tubs filled with 2 cm fine (<200μm) sieved sediment and filtered sea water (approximately 100–200 individuals). These tubs were placed in either −1.5°C or +4°C walk-in environmental chambers (three replicate tubs per temperature) with gentle aeration in each. Once a week, seawater was changed in each tub, and the worms were fed 1 gram of ground Tetramin®. No mortality was evident in the cultures and worms maintained normal feeding and tub building activities. Worms were maintained under these conditions for 4 weeks. Physiological rate measurements were made at 0, 2, and 4 weeks. Methylation profiling was executed on worms at the 4 week time point. Preliminary experimental work with this polychaete in previous field seasons indicated that 4 weeks at a +4°C thermal stress was sufficient time for physiological rate processes to re-stabilize at control (−1.5°C) levels. Thus, our time course was focused on events during this 4 week interval.

### 2.2. Physiological measurements

#### 2.2.1. Respiration

Fluorescence quenching of an organic ruthenium complex (Ruthenium(II)-tris-4,7-diphenyl-1,10-phenantroline perchlorate) in the presence of oxygen can be directly measured in optode devices. The application of this technique has been used for measuring small invertebrate respiration rates in a multi-well plate format (Szela and Marsh, 2005). Recently, we have been successful adding the Ru fluorophore directly in solution so that fluorescence efficiency is more uniform across the plate (Cowart et al., 2011). Individuals were aliquoted in 200 μl seawater and overlaid with heavy mineral oil into a 96-well flat bottom untreated polystyrene plate. For each assay, several blank wells with filtered seawater were also setup. Fluorescence was measured with a microtiter plate fluorometer (FLX-800, Bio-Tek Instruments) using 485 nm excitation and 590 nm emission filter sets. Data was logged using the manufacturer's software (KC4 Ver. 2.7.8). Respiration measurements were taken over an 8 h period with the fluorometer inside an incubator to maintain the experimental temperature. Rate analyses were calculated from a custom perl script that formats the data output from the fluorescent reader for input into a custom R script to run the oxygen consumption calculation using the Stern–Volmer equation (Glazer et al., 2004). Respiration rates were normalized to total DNA measurements of each individual (PicoGreen, Molecular Probes) as a proxy for cell number.

#### 2.2.2. Citrate synthase

The Kreb's cycle mitochondrial enzyme, citrate synthase, can serve as a proxy index of maximum aerobic capacity and is often considered a potentially sensitive enzymatic step for temperature adaptations (Fields et al., 2008). We used CS measurements to compare metabolic potential (citrate synthase activity) to actual rates of energy consumption, as measured by respiration. The general method for the measurement of citrate synthase is well established (Faloona and Srere, 1969), and it has been optimized for small volumes (Marsh et al., 1999; Pace et al., 2006). Briefly, worm tissues were homogenized in 100 μl of 50 *m*M ImidazoleÐCl (pH 7.5). Standard assay conditions used 250 μM DTNB (Ellman's Reagent), 500 μM oxaloacetate and 400 μM acetyl-coenzyme A in a final volume of 100 μl. Reaction rates were monitored at 412 nm in a plate-reading spectrophotometer inside an incubation chamber maintained at the experimental temperature. Imidazole is essentially the side group on the amino acid histidine, and it was used as the buffer because the protonation state of the amine nitrogen as a function of temperature closely parallels that of intracellular proteins (alpha-stat regulation), thereby ensuring physiologically realistic enzyme function across a broad range of temperatures (Fields et al., 2008). Substrate saturation curves were run to ensure V_*max*_ reaction rates were measured. Net reaction rates were calculated with a least-squares linear regression analysis of absorbance against time. Citrate production is calculated from the DTNB extinction coefficient and given as pmol citrate larva^−1^ min^−1^.

### 2.3. DNA methylation

On the 28th day of the temperature exposure, two replicates of 20 adults each were collected and pooled for genomic DNA extraction. DNA was isolated using a standard cetyl trimethyl ammonium bromide (CTAB) protocol and purified using Qiagen's DNeasy Blood and Tissue Kit. A 10 μg aliquot of the DNA was then digested with the methylation-sensitive restriction endonuclease HpaII, in a fashion following Jelinek et al. (2012). Overall, the strategy is similar to an earlier methyl-sensitive amplified fragment length polymorphism assay (MSAP) except that fragment detection is based on reconstruction of CpG site methylation computationally from NGS sequence read data.

Once digested, DNA was washed with Qiagen's QIAquick PCR Purification Kit and sheared to a median size of 300 bp using the Covaris AFA technology. DNA libraries were prepared using Illumina's Genomic DNA Sample Prep Kit, and 50-cycle single-read sequencing was performed on genomic DNA libraries using Illumina's sequencing by synthesis (SBS) technology on a HiSeq2500 (Delaware Biotechnology Institute, University of Delaware).

The HiSeq sequence reads were processed using a custom bioinformatic pipeline and proprietary software platform. The workflow is managed down a decision tree from raw read QC to final graphic analysis of methylation profiling differences between the experimental treatments. The novel platform performs the following tasks: (1) quality control to filter sequence tags for a designated threshold of confidence and length, (2) isolation of informative target sequence reads, (3) sequence compression to reduce complexity, (4) contig assembly using velvet, (5) CpG quantification for methyl-cytosine site distributions, (6) methylation profiling comparison between treatment groups, and (7) output of data plots and analyses. This software platform consists of a series of python scripts optimized for distributed processing on a computer cluster. The software and algorithms are proprietary as described in the Disclosure Statement. All sequence data reported here are publicly available through the Sequence Read Archive of the US National Center for Biotechnology Information (Accession #SRP040946) or they can be obtained by contacting the lead author.

## 3. Results

### 3.1. Physiology

**Figure 1:**
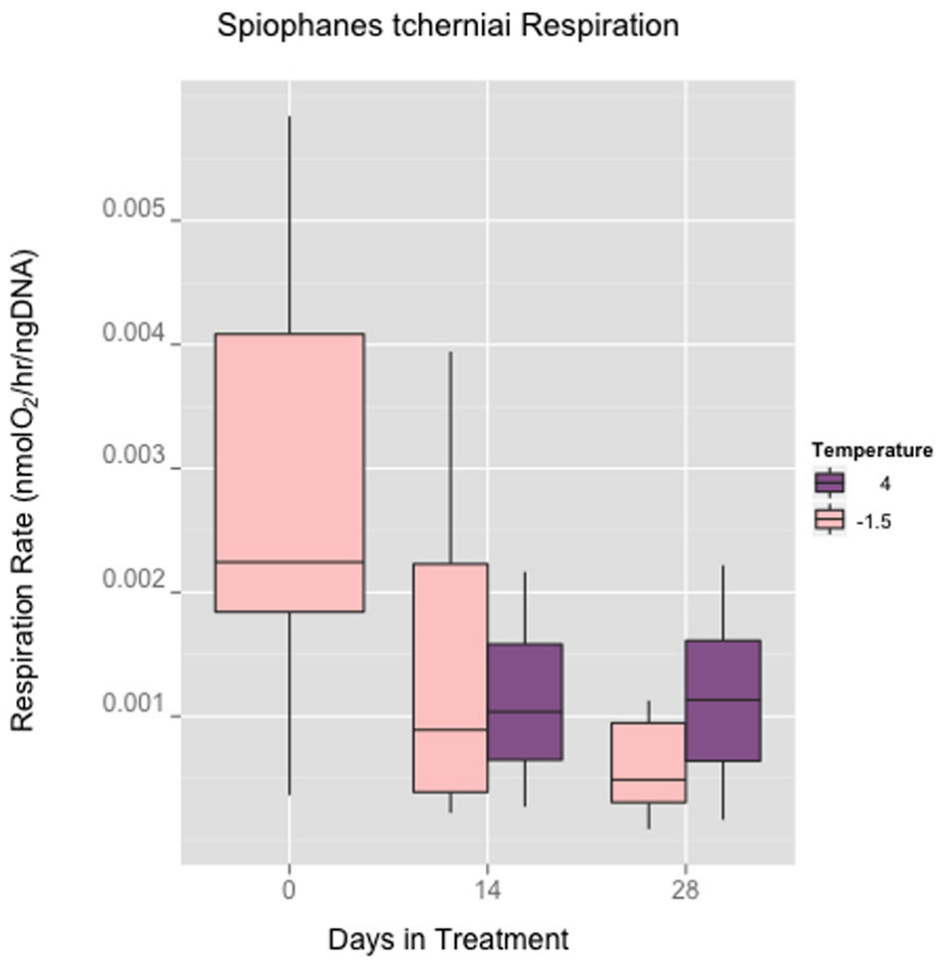
***Spiophanes tcherniai* oxygen consumption rates normalized to DNA mass in maintained for 4 weeks at −1.5C°C and +4°C**.

Field collection of adult worms requires scooping sediment up into buckets while SCUBA diving, returning the buckets to the lab, sieving the sediment to isolate adult worm tubes, and then transferring those tubes into fresh culture trays in running seawater. In Figure 1, the mechanical agitation from the collection and handling may be apparent in the wide variance in respiration rates that were measured on the 1st day that the experimental culture trays were setup. During the 4 week time course of the study, respiration rates in the control and the +4 treatment group were lower with reduced inter individual variance. Individual oxygen consumption rates were not statistically different at the 4 week sampling point (ANOVA, *n* = 23).

**Figure 2:**
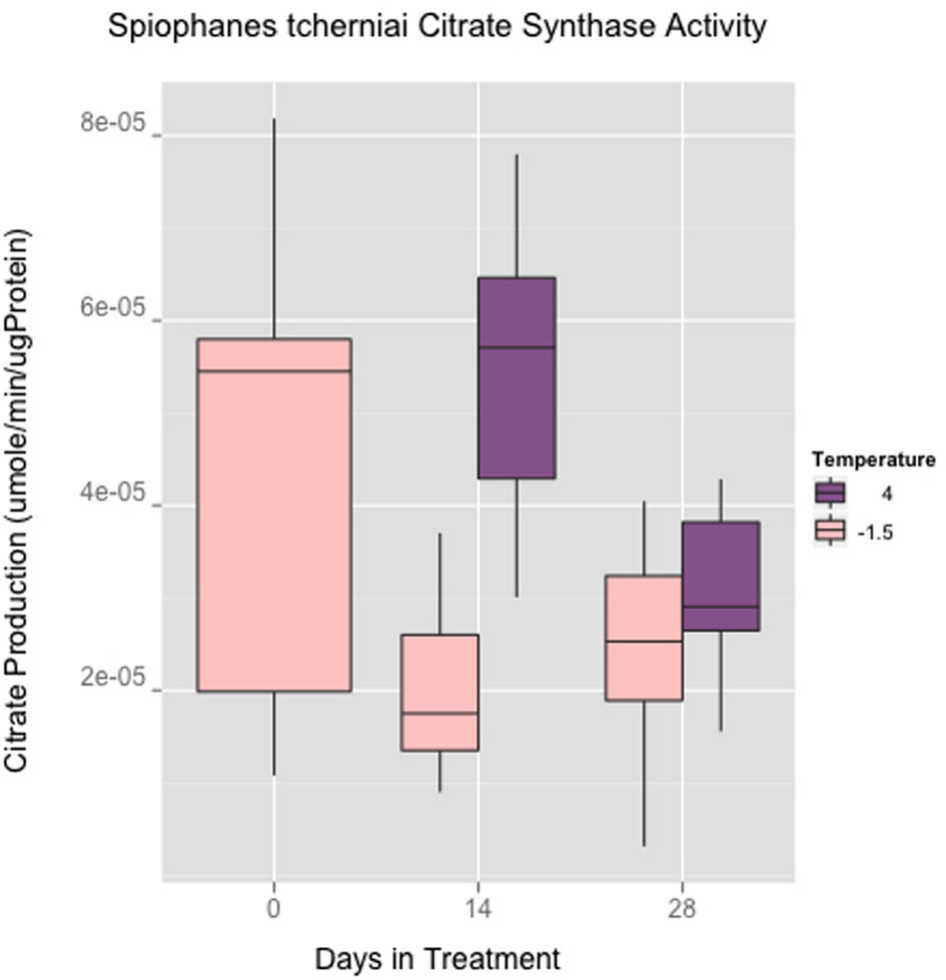
***Spiophanes tcherniai* maximum catalytic rates of citrate synthase normalized to protein mass in worms maintained for 4 weeks at −1.5°C and +4°C**.

The maximum specific activity for citrate synthase (CS) reveals a wide inter-individual variance at the onset of the experiment. Within the first 2 weeks of culturing, this variance decreases (Figure 2). There is a noticeable decrease in the mean citrate V_*max*_ at day 14 in the −1.5°C control cultures, in contrast to a maintenance of the same mean levels between day 0 and 14 in the +4°C cultures. This represents a temporal pattern in shifting means and variances suggestive of a temperature induced response between −1.5°C and +4°C cultures on day 14. However, CS V_*max*_ treatment means were not statistically different at day 28 (ANOVA, *n* = 29).

### 3.2. Bioinformatics

Two replicate gDNA samples were processed for each temperature and sequenced in separate flow cells on the HiSeq2500. Sequence reads were first processed independently for each replicate sample, but the final analysis presented here combined the raw sequence reads from replicates and processed them together. In Table 1, informatic statistics related to the sequence analysis are presented. The assembler velvet (v1.1) was used with a 21 kmer value (after optimization from 19 to 35). Note that the removal of non-informative sequence reads from the analysis results in short contigs that are concentrated around CpG repeat domains. Thus, the assembly statistics do not match results that a normal full genomic DNA assembly would produce.

**Table 1:** **Sequence and assembly informatics**.

	**−1.5°C**	**+4°C**
**SEQUENCING**		
Total reads	7,757,988	7,729,833
Reads assembled	1,495,960	1,567,432
Assembled contigs	493,107	516,081
Coverage	5.72x	5.45x
Contig n50 length	51	50
Contig max length	469	279
**CpG PROFILES**		
Total recovered CpG sites	118,651	119,550
Methylated CpG (MET)	105,399	115,910
Unmethylated CpG (UMT)	6,637	2,282
Mixed CpG (MIX)	6,475	1,231
Unkown CpG status	140	129
**CpG COMPARISON**		**−1.5°C vs. 4°C**
Matched CpG sites		28,626
Unchanged CpG status		25,559
Changed CpG status		3,067

Overall, most of the CpG sites reconstructed in the assembled contigs were methylated: 89% and 97% in −1.5°C and +4°C gDNA samples, respectively. This number is not to be confused with a percent methyl-cytosine composition measurement. Here, the fraction is essentially showing C(5mC)GG counts relative to total CCGG counts. Of the almost 120 k CpG sites recovered in the assembled contigs in each temperature, only 28 k CpG sites were matched to the same genomic contigs between treatments (23%). Of these matched CpG sites, only 3067 (11%) evidenced a change in methylation state between the temperature treatments. The remaining 25,559 sites did not vary (remained unchanged) after 4 weeks in different temperature regimes. All of the following CpG profile analyses are based on the 3067 sites that did change during the experiment, however, this response is limited to a discrete fraction of the total CpG sites recovered in the NGS sequence reads. About 90% of the CpG sites matched between the treatments had the same unchanged methylation status.

### 3.3. CpG methylation profiles

**Figure 3:**
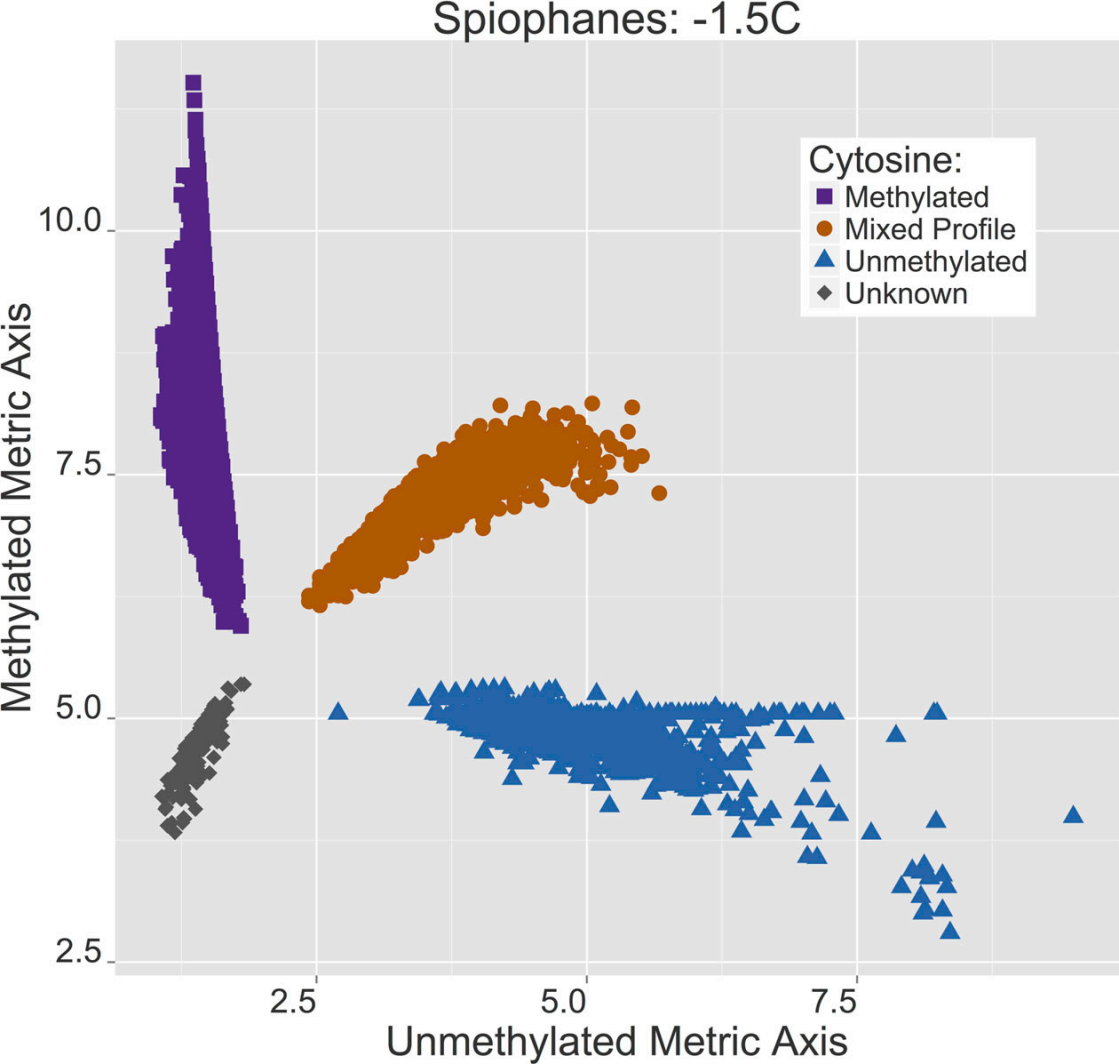
***Spiophanes tcherniai* methylation score profile for the −1.5°C worms**. Each point represents a single CpG site that has been scored for a proportional representation of methylated and non-methylated copies in these samples, with these two variables plotted on each axis. Points are divided into four groups: purple squares = methylated sites, brown circles = mixed methylation sites, blue triangles = unmethylated sites, gray diamonds = unknown or unresolved sites.

**Figure 4:**
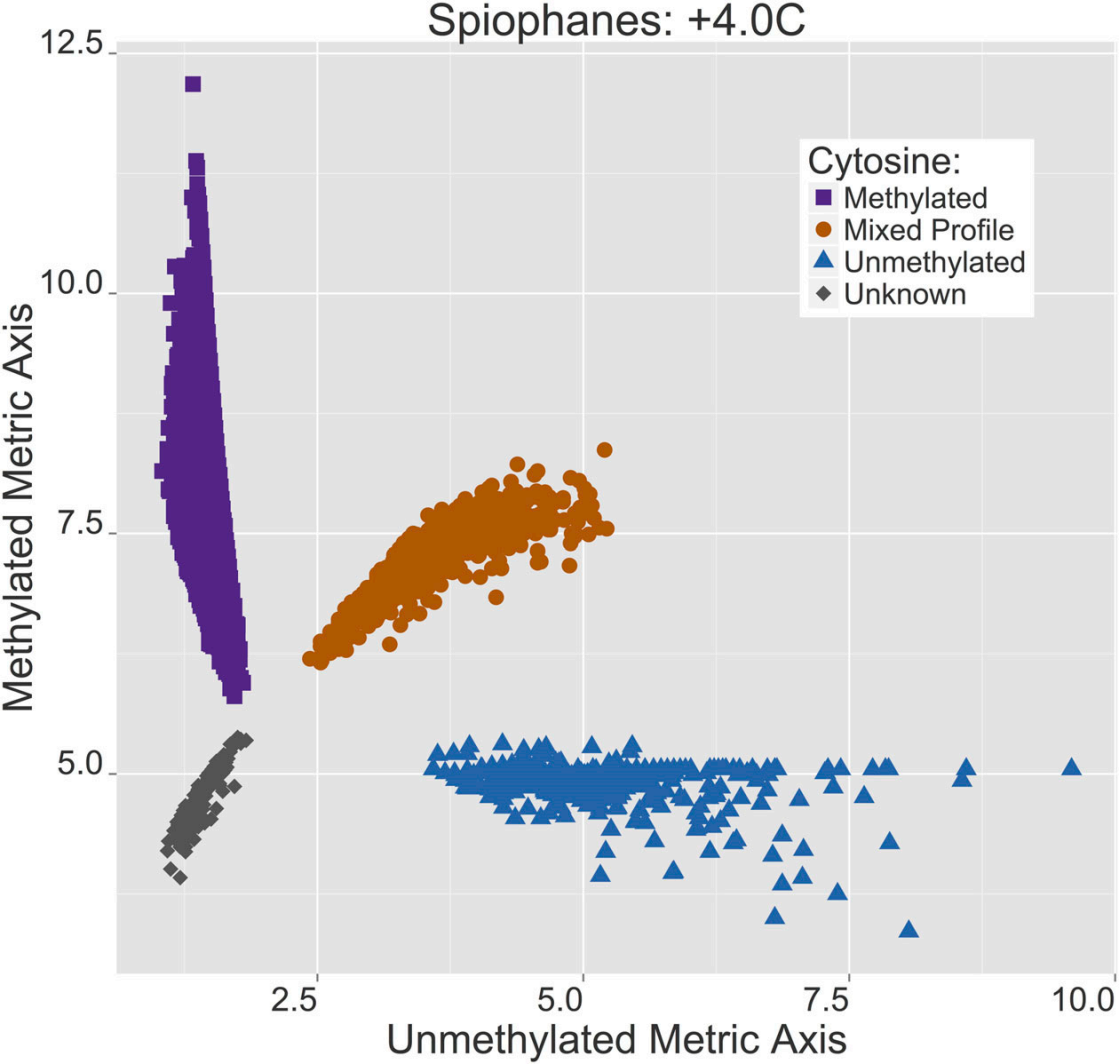
***Spiophanes tcherniai* methylation score profile for the +4°C worms**. Each point represents a single CpG site that has been scored for a proportional representation of methylated and non-methylated copies in these samples, with these two variables plotted on each axis. Points are divided into four groups: purple squares = methylated sites, brown circles = mixed methylation sites, blue triangles = unmethylated sites, gray diamonds = unknown or unresolved sites.

All of the CpG sites resolved in the filtered assembly have two metric score values corresponding to the relative count of methylated and unmethylated (5 mC) scores at the internal cytosine in a “CCGG” motif. Plotting these derivative scores locates each CpG site within a 2D area associated with high methylation (most of the copies of that contig in the sample were methylated), low methylation (most of the copies of that contig in the sample were unmethylated), or equivalent methylation. Although these designations arise from the metric analysis in the 2D plane, at this point a qualitative review of CpG sites in the assembled fragments suggests greater than 75% and less than 25% boundaries for methylated and unmethylated groupings. CpG sites for which a status determination could not be made (mostly because coverage was too low, <5*x*) are indicated as “Unknowns” (Figure 3). A complementary spatial distribution plot of CpG methylation for the +4°C worms is shown in Figure 4. All of the CpG sites plotted in Figure 3 are also plotted in Figure 4 such that the methylation status at each CpG site can be compared between treatments (*n* = 3067).

**Figure 5:**
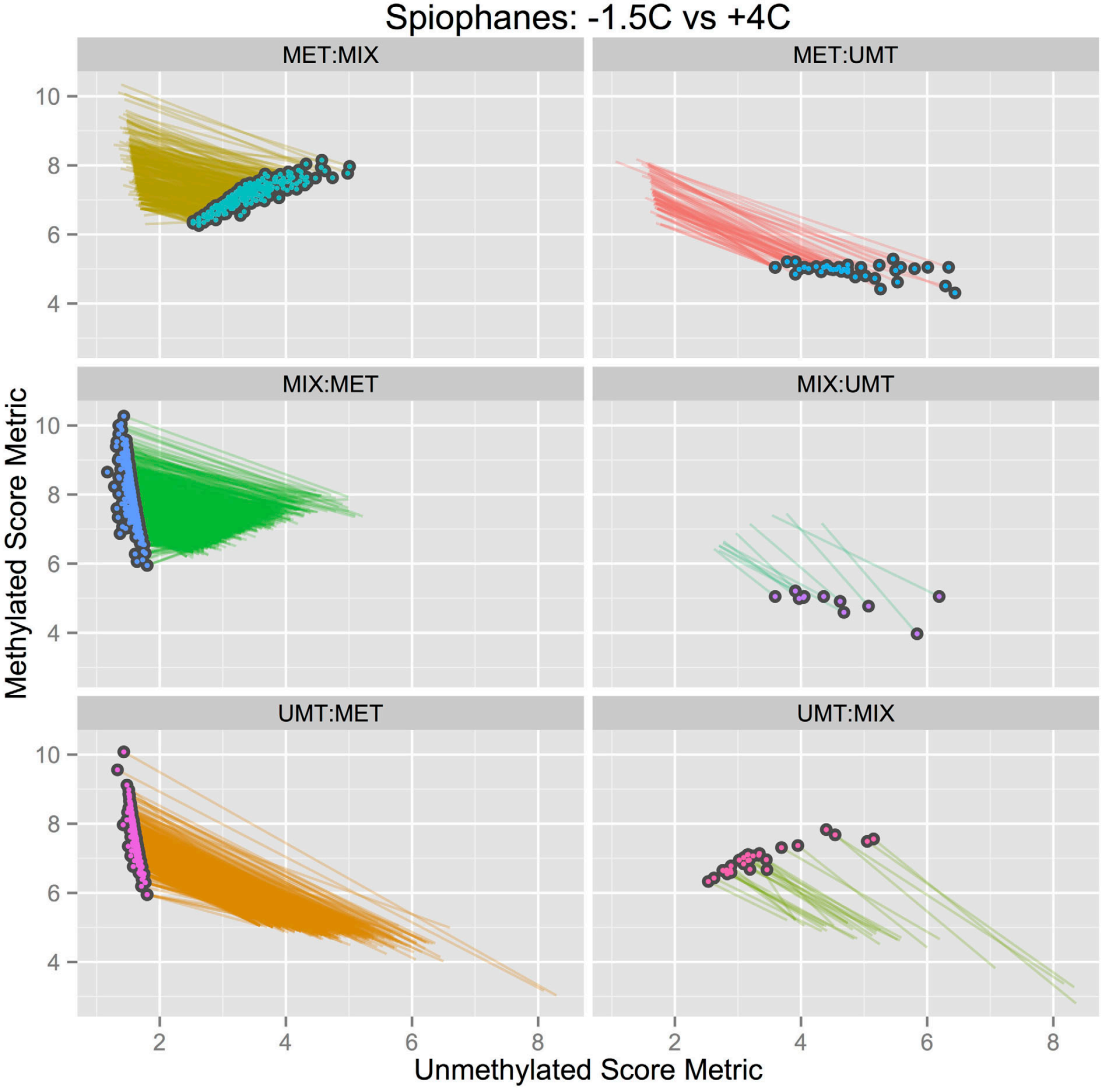
**Total methylation dynamics**. Data for each CpG site in Figures 3, 4 are now linked by a line and separated into six shifts (each panel) to reveal the pattern of shifts identified in the experiment. Each point on the plot represents a single CpG site that has evidenced a change in methylation state during the experiment (*n* = 3067 matched CpG sites). The circle symbols plot the quantitative state in the +4°C treatment while the line extending from each circle connects to the methylation state of the same CpG site in the comparative −1.5°C control treatment.

If one were to overlay the two Figures 3, 4 and draw lines to connect the same CpG site in both treatments, the comparative idea would be clear, but the resulting graph would be indecipherable. In Figure 5, this idea of overlaying the +4°C and −1.5°C data with a line for each CpG site connecting its methylation state in each treatment has been simplified by pulling the data apart into six panels, one for each potential shift. These shifts are (read *x*-to-*y*): MET:MIX (partial methylation loss), MET:UMT (full methylation loss), MIX:MET (partial methylation gain), MIX:UMT (partial methylation loss), UMT:MET (full methylation gain), UMT:MIX (partial methylation gain).

Here, a striking difference in methylation dynamics is evident between the panels. The number of CpG sites that change between treatments is shown in Table 2, where the two largest shifts between −1.5°C and +4°C are in mixed to methylated sites and unmethylated to methylated sites. In the panel plots (Figure 5), it is important to note that the length of the line for a CpG site in a panel is indicative of the strength or weight of the methylation shift. Basically, a change from 100% of a CpG site copies being unmethylated at −1.5°C and then 100% of them becoming methylated at +4°C (UMT:MET) is a greater cellular response than 50% of a CpG site copies being unmethylated at −1.5°C and then the remaining 50% become methylated at +4°C (UMT:MET). In Figure 5, one can see the type of shifts that are evident and the magnitude of those shifts. The overall pattern that emerges here is that there are two dominant methylation gains occurring (MIX:MET, UMT:MET), while only the partial methylation loss in MET:MIX is high in terms of number of CpG sites.

**Table 2:** **Directional methylation changes**.

**CpG @ −1.5°C**	**CpG @ +4°C**	**Observed counts**
MET	MIX	326
MET	UMT	73
MIX	MET	1,742
MIX	UMT	11
UMT	MET	886
UMT	MIX	28

*These values are the number of points in each panel plot in Figure 5*.

**Figure 6:**
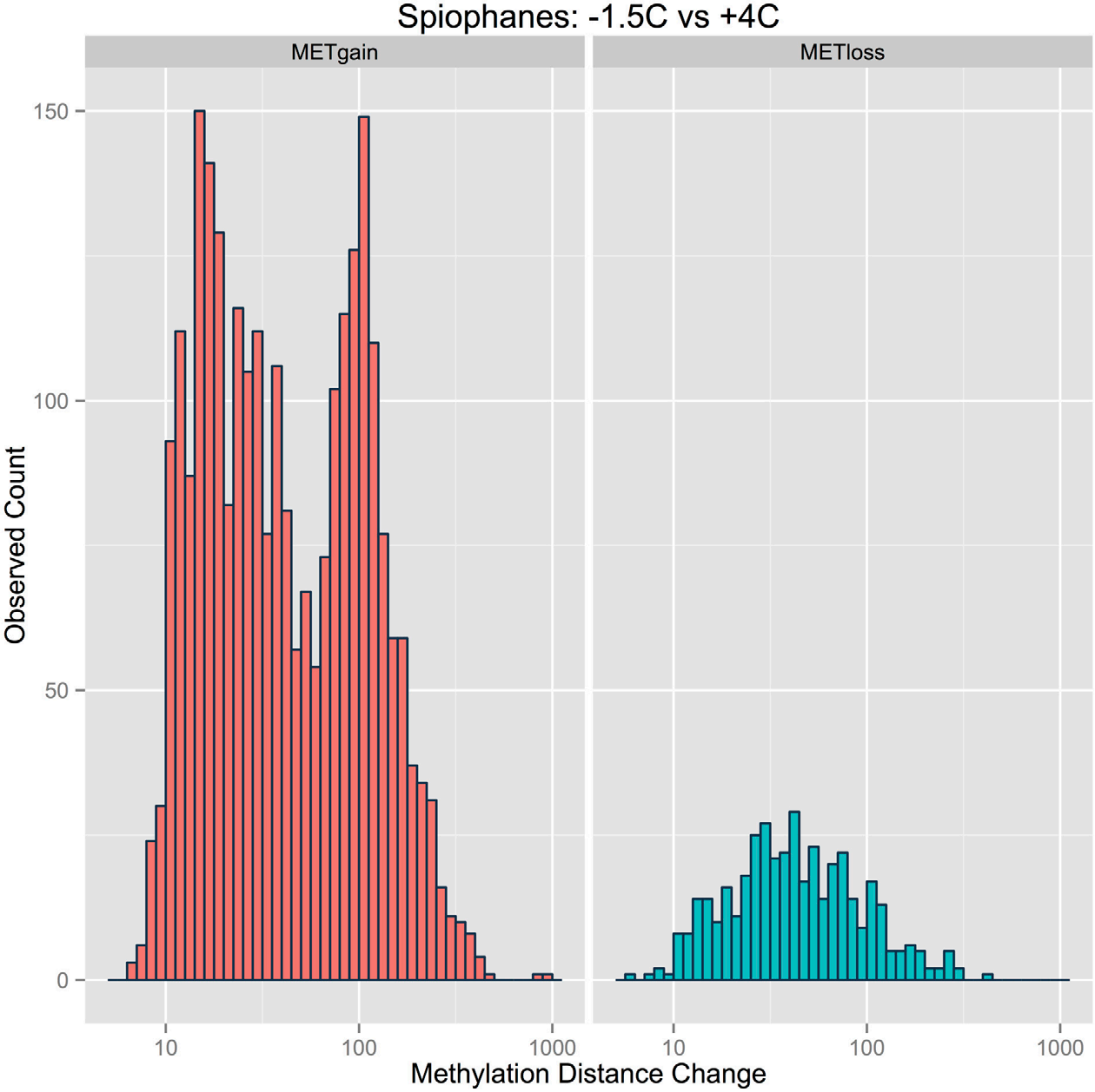
**Distributions of the magnitude of the methylation changes for each CpG site divided into gain and loss components**.

The length of the lines plotted for each CpG site in Figure 5 are essentially a representation of the magnitude of a methylation change across all copies of a CpG site in a sample. In Figure 6, frequency distribution plots are shown for these magnitude vectors divided into “Gain” and “Loss” processes. Overall, there is a striking difference in the “Gain” and “Loss” distributions in terms of both the numerical number of events and their magnitude. The observed counts here reveal the degree to which points and lines are plotted over one another in Figures 3, 4, and 5. The “Gain” distribution is clearly bimodal as a result of the strong *Full* methylation increases (Figure 5, panel UMT:MET) and the strong *Partial* methylation increases (Figure 5, panel MIX:MET).

**Figure 7:**
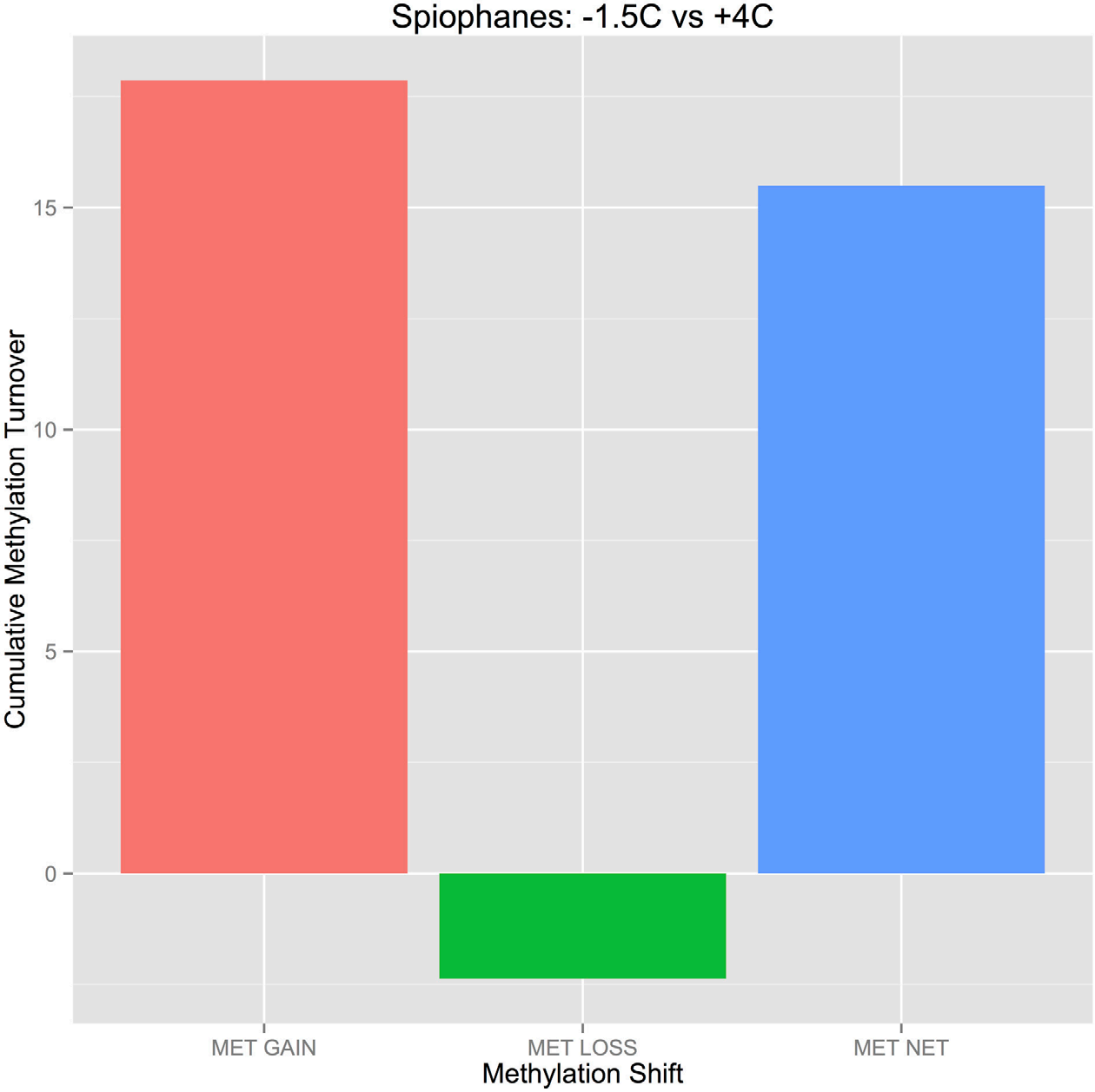
**Total magnitude of CpG site-specific changes in response to +4°C temperature treatment**. Bars present methylation gain, methylation loss, and the net methylation change among all CpG sites in response to warmer seawater temperatures (as a relative metric, based on the magnitudes of observed changes.

The magnitude scores for each CpG site methylation change in Figure 6 can be quickly summed into “Gain” and “Loss” components, and then summed to obtain a “Net Methylation Change” (Figure 7) The worms cultured at +4°C for 4 weeks demonstrably evidence a large increase (net gain) of site specific CpG methylation status relative to −1.5°C control cultures. Of the total magnitude of observed methylation changes, 88.5% were methylation gains.

## 4. Discussion

### 4.1. Temperature

The stenothermal habitat of McMurdo Sound evidences a nearly constant temperature of −1.86°C throughout much of the year, with the exception of a short, seasonal increase to around −0.5°C for 2–3 weeks in late austral summer (Hunt et al., 2003). In a 2 year period of continuous *in situ* measurements at the McMurdo intake jetty, there were only 8–21 days each year where water temperatures where above −1.1°C. In our experiment, we were able to maintain a −1.5°C control laboratory culture temperature in early Austral spring in the McMurdo aquarium facility. The warm temperature treatment of +4°C represents a temperature that *S. tcherniai* populations in the area have not been exposed to since the glaciation of the Antarctic continent, 14–20 million years ago.

The ability of *S. tcherniai* to quickly acclimate mitochondrial performance and organismal metabolism to the +4°C treatment is striking. There is an obvious large component of inter individual variance for time 0 measurements likely indicative of the handling stress in collecting and establishing the experimental cultures. This variance is essentially masking what a true mean for the field populations could be, and during the experimental time course, individuals in each treatment settle down in terms of metabolic variance. The equivalency in both respiration and CS between +4°C and −1.5°C after 4 weeks is the main result we are interested in pursuing: are rates equivalent because warmer water temperatures have no effect on metabolism in this worm, or are rates equivalent because worms are biochemically acclimated in some fashion?

As a first step to assess acclimation, we wanted to know if there was some measurement at a molecular level that we could make to ascertain whether or not these worms (−1.5°C vs. +4°C) were different in any way that might result in different metabolic activities while maintaining equivalent metabolic costs (i.e., oxygen consumption rate as an index of ATP turnover). Here, we focused our first efforts on DNA methylation as potential evidence for regulatory shifts that could possibly differentiate metabolic/biochemical activities between temperature treatments.

### 4.2. Bioinformatics

The analysis approach we have developed is similar to the strategy presented in Jelinek et al. (2012), where methyl-sensitive endo-restriction nucleases are utilized to digest gDNA during preparation for NGS sequencing. During sequence read processing and assembly the restriction sites are reconstructed computationally and quantitative metrics can be derived to describe restriction digest activity at individual CpG methylation sites.

Our results have been produced by a novel bioinformatic software platform guiding sequence data processing decisions and calculations from the QC filter of the raw sequence read files to the downstream generation of quantitative graphs and analyses (see Disclosure note). There are two important, novel facets of our approach to epigenomic profiling: (a) the analysis can detect mixed states of methylation prominence at individual CpG sites; and (b) the analysis utilizes two proprietary informatic metrics, both of which are continuous variables and not discrete binary “yes/no” classifications. These variable metrics allow us to quantitatively score methylation states relative to (x,y) positions on a 2D plane.

*(a) Mixed Methylation States:* Any tissue sample consists of a population of cells (100's of thousands). All genome copies present in a sample will not be 100% identical in terms of site-specific CpG methylation. The unique approach developed here can identify mixed patterns of methylation state (where some fraction of CpG(i) is methylated and some are unmethylated). This is critical when comparing samples for differences in methylation patterns because CpG(i) sites that are differentially methylated between two samples are likely the informative sites that account for differences in cellular function. It is these changing or dynamic CpG sites that are essential to identify and describe in terms of their potential contribution to altering functional biochemical and physiological phenotypes.

*(b) Quantitative Metrics:* The spatial location on a 2D plane using two continuous, quantitative metrics, allows us to readily calculate a strength vector for quantifying the magnitude of CpG-specific methylation shifts. Figure 5 presents a comparative plot using a line to connect the CpG(i) states between the −1.5°C and +4°C treatments. A distance metric for this separation between points serves as an intuitive metric for the magnitude of any given methylation change. This quantitative discrimination is critical for ranking the relevance of total changes observed.

### 4.3. DNA methylation

After 4 weeks of living at +4°C, *S. tcherniai* exhibit large shifts in DNA methylation patterns in direct comparison to control worms maintained at −1.5°C. Overall, there is a predominance of methylation gains at specific CpG sites. This is clear in the aggregate summary histogram in Figure 7, the methylation magnitude of gains and losses in Figure 6, and the single CpG tracking lines in Figure 5. The large numerical shift in the UMT to MET state (Figure 5, lower left panel) represents the archetypical, binary response that one tends to focus on when thinking about epigenetic shifts. Here, all CpG-site copies (gDNA sample has 100's thousands of genome copies from every cell in the sample) were unmethylated at day 28 in worms living at −1.5°C while all corresponding CpG-site copies in the +4°C worms were fully methylated. It is easy to think of this as an on/off switch response.

The ability to score a CpG site as being fractionally methylated among gDNA copies in a sample opens up a new avenue for assessing DNA methylation responses to changing cellular conditions. By quantitatively distinguishing a “mixed” population of copies of one CpG site, we can begin to identify dynamic, non-binary, continuous-response reactions in DNA methylation intensity or density (average change per copy). Looking at the MET:MIX and MIX:MET panels in Figure 5, we see a dynamic balance between CpG sites that switch between states of 100% fully methylated across all gDNA copies to 50% mixed states. We can conclude that these CpG sites are dynamic and that a 50% methylation position represents a response to shift in some cells, but not others, resulting in a mixed population of CpG specific states within a gDNA extract.

This differential or dynamic response raises the obvious question of how cell-type may impact gDNA methylation profiles when whole organisms are sampled. Tissue heterogeneity is not a new problem to genomic profiling, where even needle biopsies are large enough to include cell to cell variance in genome states. It is likely that the mixed population measurements result from gDNA originating from different tissues within an adult worm. Here, it is important to note that we achieve a large degree of numerical differentiation with our analysis and that within a group designation, such as MET, the *y*-axis methylated score metric still ranges almost 2-fold, from 6 to 10 metric units. We are pursuing further refinements to assess if this score range may be further used in future analyses to classify gDNA copies into different cell types.

## 5. Conclusion

We have performed an epigenetic profiling analysis on a marine invertebrate that has no prior molecular genomic resources available. We have shown that DNA methylation responses to small changes in temperature in this Antarctic worm are dominated by gains in 5 mC abundance. However, even though the result is informative, the problem now is that this information would be more useful and insightful if we could map those CpG shifts back to defined gene loci. We've attempted to utilize the polychaete *Capitella teleta* genome resources available (Joint Genome Institute, PI E. Seaver). Unfortunately, work to derive a genome fragment assembly for *S. tcherniai* is still in progress. We are currently sequencing deeper in order to assemble larger contigs covering sufficient protein coding domains so that those contigs can be confidently mapped to *C. teleta* loci. At this point, we know the next step to putting this response in a functional context is going to require more gDNA work. This is the challenge of working with non-model organisms.

An essential component of the definition of any living system is the environment that surrounds it, and to which the organism is intimately connected. We could describe the polychaete *S. tcherniai* as an open system constantly engaged in complex interactions with its surroundings. The separation of animal from environment is tenuous because of the perpetual exchange of physical, chemical, mechanical, and biological information between them (Alberghina et al., 2009). Many of these external forces can initiate internal, cellular chromatin remodeling resulting in an epigenome that is continuously modified in response to environmental factors, like food availability or physiological stress (Vinci, 2011).

As more epigenetic work shifts from model systems to focusing on organisms that live in natural environments, we are beginning to understand the tight associations that can exist between an organism's epigenome and it's habitat (Elango et al., 2009; Gavery and Roberts, 2010; Okamura et al., 2011; Gmez-diaz et al., 2012; Falckenhayn et al., 2013; Glastad et al., 2013; Wang et al., 2013). In this paper we have shown that metabolic rates in *S. tcherniai* return to normal within 4 weeks following a high-temperature stress, and that shifting patterns of DNA methylation arise during this time course. Work continues to identify the degree to which those methylation shifts may play a role in the regulation of energy metabolism in this Antarctic polychaete.

## Funding

This work was made possible through a grant from the National Science Foundation to Adam G. Marsh (Office of Polar Programs, #0944557), and with National Science Foundation support to Adam G. Marsh for the commercial development of the software (Innovation Corps program, #1355306).

### Conflict of interest statement

The software platform designed for processing DNA methylation profiles from NGS sequence data is proprietary and covered by a provisional patent application filed by the University of Delaware, which is currently being considered for an exclusive license agreement by a company co-founded by Adam G. Marsh. These activities are supported by an Innovation Corps Grant from the National Science Foundation as noted below.
